# Association of Surgical Adhesive and Glue System With Incidence of Allergic Dermatitis Post Unilateral and Staged Bilateral Total Knee Arthroplasty

**DOI:** 10.7759/cureus.58827

**Published:** 2024-04-23

**Authors:** Enji Ali, Asim M Makhdom

**Affiliations:** 1 Orthopaedic Surgery, Dr. Samir Abbas Hospital, Jeddah, SAU; 2 Orthopaedic Surgery, King Abdulaziz University, Jeddah, SAU

**Keywords:** hypersensitivity, allergies, adhesives, arthroplasty, knee

## Abstract

Background

Skin closure is an important step in total knee arthroplasty (TKA). Several techniques are described in the literature with emerging interest in skin adhesives. This study aims to highlight the adverse reactions related to skin adhesives and to identify potential risk factors.

Methods

A retrospective review of 250 patients (295 knees, 45 being staged bilateral TKA) who underwent TKA was conducted at our facility. All patients underwent the same perioperative protocol with application of surgical glue over the wound edges. Postoperative adverse reactions were classified as allergic dermatitis (AD), cellulitis, and superficial infection.

Results

Incidence of adverse reactions in the form of AD was noted in 4.8% of patients. Demographics and comorbidities had no influence on this occurrence (p>0.05). However, in patients who had bilateral staged TKA, AD manifested after performing the second contralateral TKA in 22% of patients. This incidence of AD was significantly higher when compared to those who had single unilateral TKA (p =0.001)

Conclusion

The theory of allergic sensitization may explain the higher incidence of AD in bilateral staged TKA due to prior exposure to glue components in the first knee; thus, we recommend avoiding its use if there was previous exposure.

## Introduction

Skin closure is the last important step of the total knee arthroplasty (TKA) procedure. Several closure techniques have been reported including skin sutures, barbed sutures, staples, and adhesive materials [1]. The choice of optimum skin closure technique has not reached a consensus and is an ongoing focus of investigation. Surgical site infections (SSI) are a morbid complication, especially post total joint arthroplasty (TJA) [2]. The literature supports the relationship between prolonged wound drainage and increased risk of SSIs [3], and thus, several dressing techniques such as negative pressure dressings [4,5], occlusive dressings, antimicrobial dressings [6], and skin adhesives have been proposed with variable reported outcomes [7].

Surgical glue has been successfully used since 1949 for the closure of traumatic lacerations. Its success is attributed to a decrease in postoperative wound leakage and providing a strong microbacterial barrier by polymerizing in the presence of fluids to keep the skin edges together [8]. Additional advantages include lack of skin toxicity, decreased risk of wound edge ischemia, improved cosmesis, and reduced operative time [9]. Recently, interest has risen in using it for wound closure in TJA. While the surgical glue system provides many benefits, it has been noted that a percentage of patients develop wound complications related to it. These complications can be categorized as allergic dermatitis (AD), wound dehiscence, cellulitis, and superficial infections. AD is noted to occur most commonly to the chemical 2-octyl cyanoacrylate and is in the form of blisters and an erythematous rash around the wound edges [10,11]. A similar component has been used to glue eyelashes and has been found to cause contact dermatitis by Sato et al. [12]. They attributed the development of AD to previous sensitization to the implicated compound in the glue or the presence of cross-reactivity to a previously used material [12].

In TKA patients, a clinical difficulty may be faced in differentiating between AD and superficial wound infection, and the skin breakdown too can be a portal for deeper infections. There is no data noting the incidence and significance of these reactions post TKA. The primary aim of this study was to report the incidence of wound complications while using surgical glue (Dermabond Advanced Topical Skin Adhesive; Ethicon, Inc., Raritan, New Jersey, United States) as a method of skin closure post TKA. The secondary aim was to identify possible risk factors that lead to such complications.

## Materials and methods

This study was approved by King Abdulaziz University Hospital Unit of Biomedical Ethics (approval number: 232-23). The electronic records of all consecutive patients who underwent TKA from August 2021 to December 2022 at King Abdulaziz University Hospital, Jeddah, Saudi Arabia, were retrospectively reviewed.

During the study period, 260 patients underwent TKA. Inclusion criteria were patients who underwent TKA and had severe symptomatic arthritis. Exclusion criteria included patients known to have an allergy to skin adhesives, those with bleeding diathesis, a previous incision at the surgical site, revision surgery, and those who are immunosuppressed (due to renal or hepatic impairment, poorly controlled rheumatological disorder). Thus, 10 patients were excluded. This left 250 who were included in our study. Of these, 45 had bilateral staged TKA and performed their second TKA with a minimum duration of six weeks in between each TKA.

Patients' basic demographics, age, gender, body mass index (BMI), medical comorbidities, and American Society of Anesthesiologists (ASA) grade were recorded. Any history of drug or food allergies was also collected. 

All patients received cefazolin dose 30 minutes prior to the incision and at least two additional doses post-surgery; 1 gram (g) of intravenous tranexamic acid was given prior to the incision and another 1 g was given at the time of closure. All primary TKA procedures were performed by the senior author (AM) who is an Arthroplasty Fellowship-trained surgeon. The arthrotomy was closed using barbed sutures (Stratafix size 1; Ethicon, Inc.), followed by Vicryl 0 and 2-0 (Ethicon, Inc.) for the subcutaneous tissues. The skin was closed using subcuticular Monocryl 3-0 (Ethicon, Inc.) and a layer of Dermabond (Ethicon, Inc.), composed of a monomeric (2-octyl cyanoacrylate) formulation and the colorant D & C Violet # 2, applied directly on the wound without application of the mesh component. Adhesive strips were then placed perpendicularly on the wound followed by a waterproof dressing. No drain was used in all patients. 

Patients were seen one week postoperatively whereby the dressing was removed for the first inspection of the incision to look for any erythema, leakage, dehiscence, or skin reactions. Any adverse skin reactions were recorded postoperatively and are classified as AD, wound dehiscence, cellulitis, and superficial infection. The difference between AD and a superficial infection was ascertained by clinical examination and confirmed by laboratory markers (C reactive protein (CRP) and erythrocyte sedimentation rate (ESR)). If the CRP was elevated more than 100 mg/L during the early postoperative period, a joint aspiration was performed to rule out infection.

Statistical analysis

Means, ranges, and standard deviations were used for descriptive statistics. The chi-square test was used to compare proportions between categorical variables, and unpaired t test was used to compare two means. Additionally, logistic regression was performed for multivariate analysis and included all the study variables to account for any confounding factor that could have affected the incidence of allergic dermatitis following TKA. P-value < 0.05 was considered significant, and CI was set at 95% to consider a statistically significant difference. IBM SPSS Statistics for Windows, Version 20.0 (Released 2011; IBM Corp., Armonk, New York, United States) was utilized for the statistical analysis.

## Results

This study included 250 patients (295 knees). The majority were females (81.2%), and the mean age was 66.58 ±7.87, (range, 48-91). Single TKA was performed in 82.0% (n=205) of the cases, while 18.0% (n=45) had bilateral staged TKA. The second TKA in those who had bilateral staged TKA was performed within six weeks to six months after the first TKA. In one patient (out of the 45) the second TKA was performed after one year. Demographic characteristics of the patients as well as their comorbidities are given in Table 1.

**Table 1:** Sociodemographic characteristics and comorbidities (N=250) Data expressed as number and percentage except for Age and BMI, which are expressed as mean ± SD

Characteristics	Number	Percentage
Sex		
Male	47	18.8%
Female	203	81.2%
Comorbidity presence		
Yes	190	76.0%
No	60	24.0%
Diabetes		
Yes	103	41.2%
No	147	58.8%
Hypertension		
Yes	122	48.8%
No	128	51.2%
Coronary artery disease		
Yes	14	5.6%
No	236	94.4%
Asthma		
Yes	15	6.0%
No	235	94.0%
Rheumatoid arthritis		
Yes	7	2.8%
No	243	97.2%
American Society of Anesthesiology (ASA) grade		
1	79	31.6%
2	161	64.4%
3	10	4.0%
Unilateral or Bilateral Total Knee Arthroplasty (TKA)		
Unilateral TKA	205	82.0%
Bilateral TKA	45	18.0%
	Mean±SD, range	
Age	66.58 ±7.87, (48-91)	
Body mass index (BMI)	32.70 ± 4.22, (21-43)	

None of our patients had postoperative drainage. However, wound complications in the form of AD occurred in 4.8% (n=12). These were noted within 7-10 days postoperatively. The relationship between different demographic characteristics and the occurrence of allergic dermatitis is given in Table 2.

**Table 2:** The effect of different variables on the incidence of allergic dermatitis ^*^ Statistical significance was set at p <0.05 Data expressed as number and percentage except for Age and BMI, which are expressed as mean ± SD

Characteristics	n (%)	Incidence of allergic dermatitis	p-value
Sex			1.000
Male	47 (18.8%)	4.3%	
Female	203 (81.2%)	4.9%	
Comorbidity presence			0.736
Yes	190 (76.0%)	5.3%	
No	60 (24.0%)	3.3%	
Diabetes			0.559
Yes	103 (41.2%)	5.8%	
No	147 (58.8%)	4.1%	
Hypertension			0.703
Yes	122 (48.8%)	5.7%	
No	128 (51.2%)	3.9%	
Coronary artery disease			0.507
Yes	14 (5.6%)	7.1%	
No	236 (94.4%)	4.7%	
Asthma			1.000
Yes	15 (6.0%)	0.0%	
No	235 (97.2%)	5.1%	
Rheumatoid arthritis			1.000
Yes	7 (2.8%)	0.0%	
No	243 (97.2%)	4.9%	
American Society of Anesthesiology (ASA) grade			0.631
1	79 (31.6%)	5.1%	
2	161 (64.4%)	4.6%	
3	10 (4.0%)	10.0%	
Unilateral or Bilateral Total Knee Aarthroplasty (TKA)			0.001*
Unilateral TKA	205 (82.0%)	1.0%	
Bilateral TKA	45 (18.0%)	22%	
	Mean ± SD	Means of allergic dermatitis	
Age (years)	66.58±7.87	65.00±7.28 (yes) vs 66.03±7.9 (no)	0.709
Body Mass Index (BMI)	32.70±4.22	33.58±2.51 (yes) Vs 32.66±4.29 (no)	0.258

Neither age, sex, ASA score, comorbidities, or BMI affected the incidence of AD. One patient had a history of food allergy (fish) and two had a history of betadine allergy; none had adverse reactions postoperatively. However, there was a significant incidence of AD in those who had bilateral staged TKA. Ten out of 12 cases of AD were in patients who underwent bilateral TKA and manifested within 7-10 days after the second TKA. This represents a 22% incidence among patients who had bilateral staged TKA (10 out of 45). On the contrary, only two cases of AD occurred in patients who underwent unilateral TKA, which only resembled 1% of that group of patients. After adjusting for multivariate analysis, developing AD was statistically significant in patients with bilateral staged TKA when compared with patients who only had one single TKA (p= <0.001, OR=46.53, 95%CI= 7.32-295.50).

**Figure 1:**
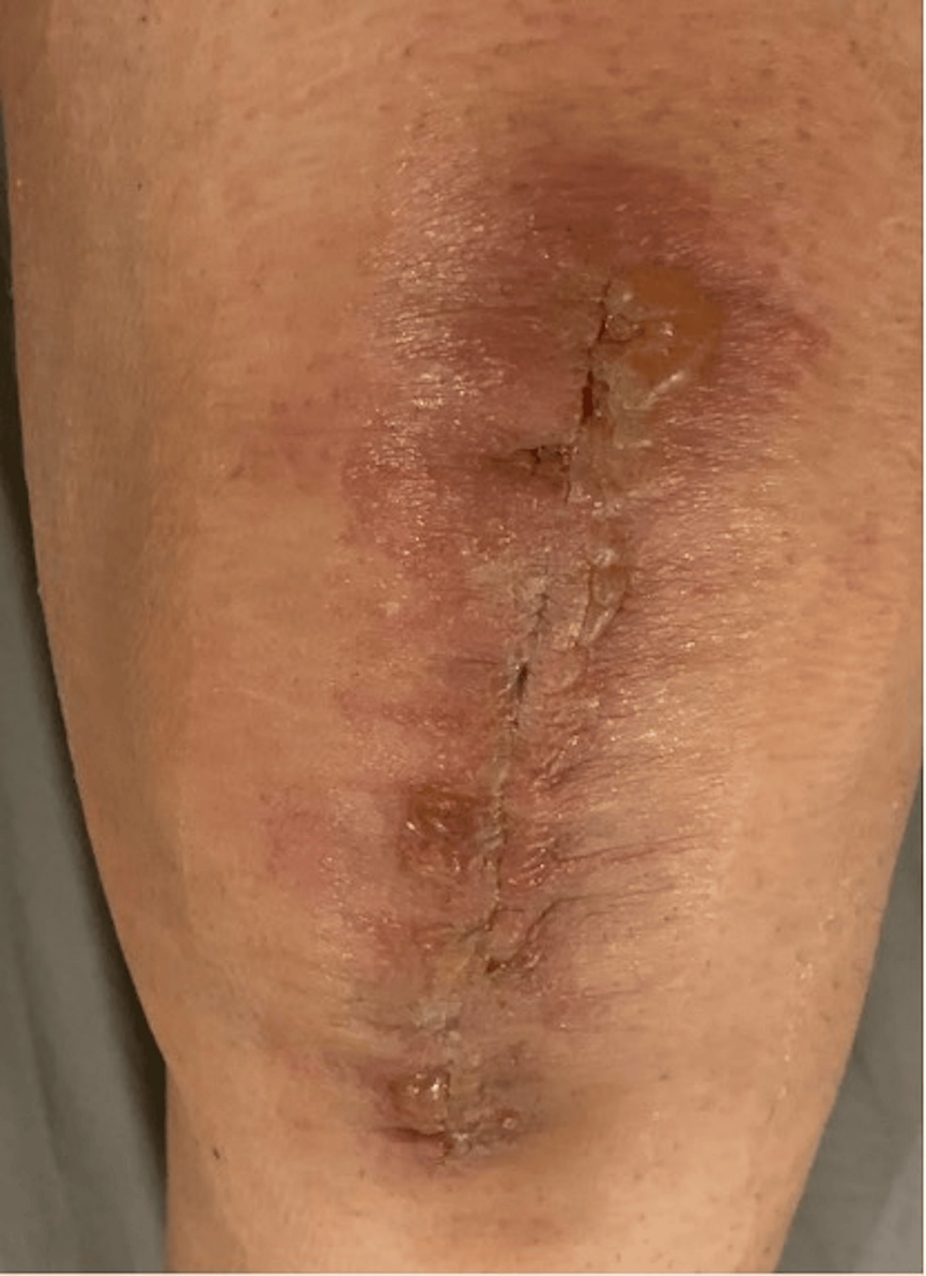
Knee incision image of allergic dermatitis one week post surgery

**Figure 2:**
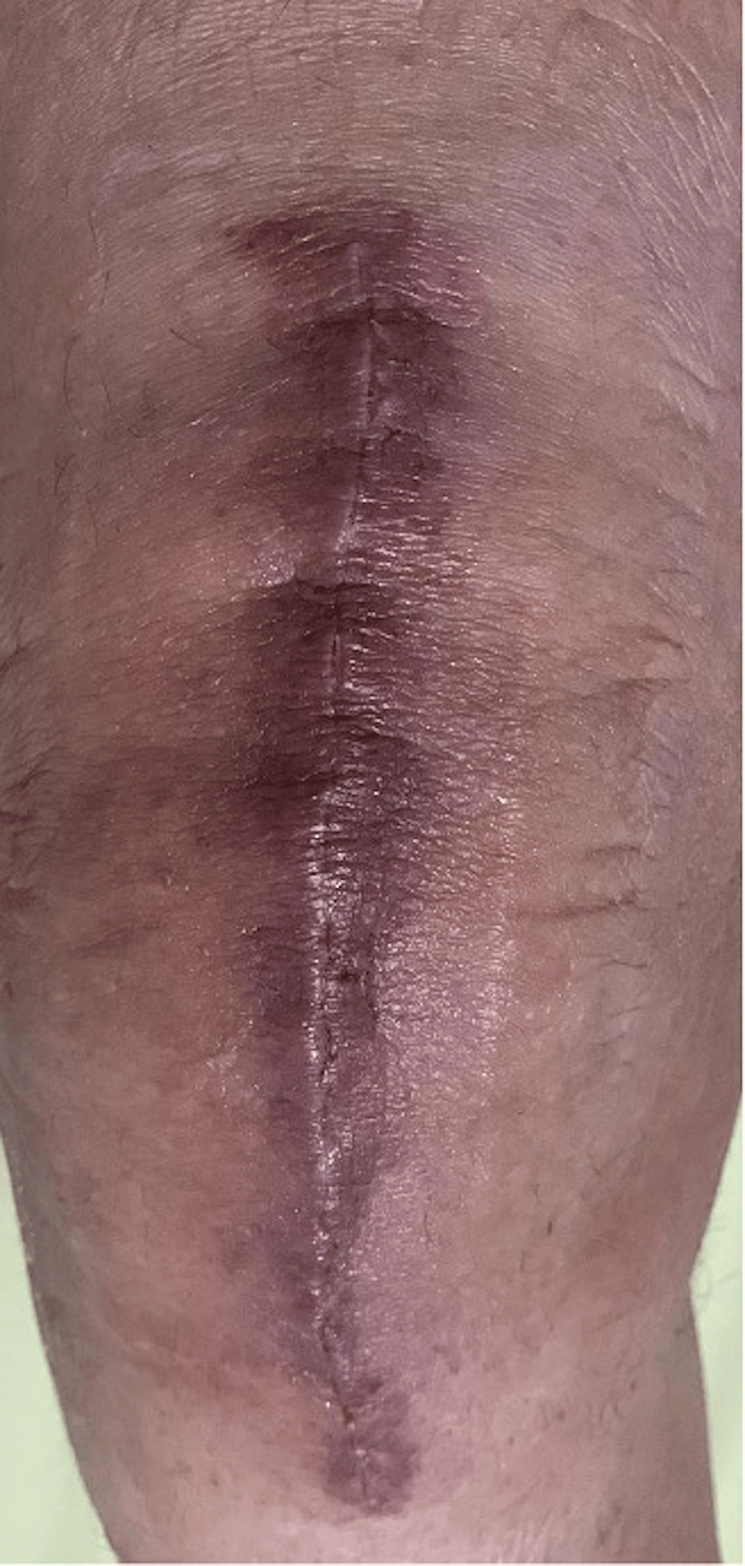
The same patient shown in Figure 1 two weeks after initial postoperative visit, showing signs of healing

All symptoms of AD in our study resolved in a period of two to three weeks by local treatment which involved the application of a topical antibiotic/steroid ointment over the affected area twice daily (Figure 1 and Figure 2).

Oral antihistamines were prescribed as well to reduce the pruritis. None of our patients developed cellulitis or a superficial infection. Deep infection was suspected in one patient and was ruled out by the results of synovial joint fluid aspiration

## Discussion

This study presents the incidence of skin complications related to Dermabond use without mesh application in TKA patients. It was noted that 4.5% of all patients developed skin allergic reactions to the adhesive material post TKA but the majority of these were in patients who had bilateral staged TKAs. Interestingly, AD manifested mainly after performing the second TKA. We believe this allergic reaction can be identified as a type IV hypersensitivity reaction secondary to re-exposure to the antigen. Most probably the sensitization occurred from the usage of Dermabond after its application during the first TKA in staged TKA cases. Patients who developed AD after the first knee surgery could be explained by prior exposure to a similar compound in other products resulting in cross-reactivity [12]. Sensitization can last up to 10 years from the last antigen exposure due to immunologic memory. A recent case report describes the development of AD after the third exposure to the adhesive system, which explains the significance of long-term immunologic memory [13]. Type IV hypersensitivity reactions are cell-mediated by CD4+ and CD8+ T cells, macrophages, and inflammatory cytokines. This results in direct tissue damage by T cells or indirectly by the release of enzymes [14]. Thus, upon re-exposure to the antigen during the second TKA or primary exposure in the presence of cross-reactivity in the case of the first knee, the immunologic memory is reactivated and a type IV hypersensitivity reaction occurs explaining the higher incidence of adverse reactions after the second TKA. 

There are several studies outlining the benefits of using adhesive glue when compared to other closure systems post TKA with only a few highlighting its’ skin complications in western patients. Chalmers et al. reported an incidence of 0.5% in a series of 6088 units of Prineo used in hip and knee arthroplasty over a period of three years [15]. A higher number was noted in knee compared to hip replacements, which the author attributes to the higher tension present on extensor surfaces such as the knee. Durando et al. reported an incidence of 2% from 915 consecutive TKA that used a skin adhesive system [16], which is similar to the incidence in the current study for patients undergoing the second knee TKA which was resolved by local treatment. A case report by Pate et al. described a severe reaction post shoulder hemiarthroplasty that necessitated debridement of the skin edges and skin grafting [17]. Furthermore, another case report identified the mesh component in Dermabond Prineo as a possible allergen in addition to the 2-octyl cyanoacrylate present in the glue [18]. However, in our study, none of the patients had mesh components, and therefore, Dermabond glue was the only possible allergen.

There are a few case series in the literature that suggest patch testing to identify those at risk of developing adverse reactions. Durando et al. note a positive patch test in relation to 2 octyl- cyanoacrylate in two out of three patients who developed a reaction post TKA [16,19]. The utility of such tests in the acute setting is not feasible as it takes five days to get the result and it’s not widely available. In our study, we evaluated the demographic characteristics (age, BMI, sex, ASA score, different comorbidities such as asthma and food allergies) of our patients to check if they pose a risk factor in developing AD and it was noted that none increased the incidence of developing AD. This is supported by a recent study that evaluated different demographic variables as possible risk factors for developing AD and noted that none of these increased the risk of developing AD [20].

The advantages of the glue adhesive system include decreased wound drainage, a strong antimicrobial barrier, and a strong wound seal, which have a strong role in reducing surgical site infections. In the current study, we found that none of our patients have had postoperative drainage. While this can be multifactorial, using a Dermabond adhesive glue probably had a significant contribution. We are currently still using Dermabond as a part of our closure technique for TKA but omitted using it for the contralateral TKA for those undergoing bilateral staged TKAs.

Limitations of this study include the retrospective nature and lack of a comparative control group. Also, this study is based on a single type of adhesive material, and extrapolation to other forms of adhesives would not be possible. However, the main strength of this study is finding a higher incidence of AD after re-exposure in patients who have had bilateral staged TKA. Additionally, only the glue component was used in our patients without the mesh, thus reducing the probability of these reactions being due to another source. To the best of our knowledge, this is the first study reporting the incidence of skin reactions post Dermabond usage in TKA in Middle Eastern patients and highlighting the high incidence of adverse reactions when patients have prior exposure to such adhesive (e.g., prior TKA).

## Conclusions

Skin adhesives provide a significant means to reduce wound drainage post TJA by forming a strong waterproof seal. Adverse reactions to skin adhesives were identified as AD in our population. The incidence of AD in patients who had single unilateral TKA was 1% but a higher incidence (22%) of AD was found in patients who had bilateral staged TKA. This study emphasizes the theory of sensitization as a cause of increased incidence of these adverse reactions in patients undergoing TKA with prior exposure to the components in the skin adhesives (in our study it was during the first TKA skin closure). Due to the high incidence of AD in these patients, we recommend avoiding the use of Dermabond in patients with prior exposure.
